# Effects of Heavy Ion Particle Irradiation on Spore Germination of *Bacillus* spp. from Extremely Hot and Cold Environments

**DOI:** 10.3390/life10110264

**Published:** 2020-10-30

**Authors:** Vincenzo Zammuto, Maria G. Rizzo, Laura M. De Plano, Domenico Franco, Salvatore Guglielmino, Maria T. Caccamo, Salvatore Magazù, Akira Fujimori, Angelina Lo Giudice, Mauro Guglielmin, Kevin Roderick McAlpin, Ralf Moeller, Concetta Gugliandolo

**Affiliations:** 1 Department of Chemical, Biological, Pharmaceutical and Environmental Sciences, Research Centre for Extreme Environments and Extremophiles, University of Messina, V.le F. Stagno d’Alcontres 31, 98166 Messina, Italy; vzammuto@unime.it (V.Z.); ldeplano@unime.it (L.M.D.P.); dfranco@unime.it (D.F.); sguglielm@unime.it (S.G.); cgugliandolo@unime.it (C.G.); 2 Department of Mathematics, Computer Sciences, Physics and Earth Sciences, University of Messina, V.le F. Stagno d’Alcontres 31, 98166 Messina, Italy; mcaccamo@unime.it (M.T.C.); smagazu@unime.it (S.M.); 3 Department of Basic Medical Sciences for Radiation Damages, Molecular and Cellular Radiation Biology Group, NIRS/QST, Chiba 263-8555, Japan; fujimori.akira@qst.go.jp; 4 Institute of Polar Sciences, National Research Council (CNR-ISP), Spianata San Raineri 86, 98122 Messina, Italy; angelina.logiudice@cnr.it; 5 Department of Theoretical and Applied Sciences, University of Insubria, Via J.H. Dunant, 21100 Varese, Italy; mauro.guglielmin@uninsumbria.it; 6 Institute of Aerospace Medicine, Radiation Biology Department, German Aerospace Center, Aerospace Microbiology, DLR, Linder Höhe, D-51147 Cologne/Köln, Germany; ralf.moeller@dlr.de (R.M.); kevin.mcalpin@dlr.de (K.R.M.); 7 Natural Sciences Department, University of Applied Sciences Bonn-Rhein-Sieg (BRSU), D-53359 Rheinbach, Germany

**Keywords:** *Bacillus*, extremophiles, heavy ion particle (HZE) radiations, nutrient germinants, spore germination, spore resistance

## Abstract

Extremophiles are optimal models in experimentally addressing questions about the effects of cosmic radiation on biological systems. The resistance to high charge energy (HZE) particles, and helium (He) ions and iron (Fe) ions (LET at 2.2 and 200 keV/µm, respectively, until 1000 Gy), of spores from two thermophiles, *Bacillus* *horneckiae* SBP3 and *Bacillus* *licheniformis* T14, and two psychrotolerants, *Bacillus* sp. A34 and A43, was investigated. Spores survived He irradiation better, whereas they were more sensitive to Fe irradiation (until 500 Gy), with spores from thermophiles being more resistant to irradiations than psychrotolerants. The survived spores showed different germination kinetics, depending on the type/dose of irradiation and the germinant used. After exposure to He 1000 Gy, D-glucose increased the lag time of thermophilic spores and induced germination of psychrotolerants, whereas L-alanine and L-valine increased the germination efficiency, except alanine for A43. FTIR spectra showed important modifications to the structural components of spores after Fe irradiation at 250 Gy, which could explain the block in spore germination, whereas minor changes were observed after He radiation that could be related to the increased permeability of the inner membranes and alterations of receptor complex structures. Our results give new insights on HZE resistance of extremophiles that are useful in different contexts, including astrobiology.

## 1. Introduction

The boundary conditions of life on Earth have been tested in different possible directions, encompassing the limits of temperature, pH, pressure, salinity, nutrients, and radiations. A great variety of terrestrial environments exhibit extremes in one or more physical or chemical conditions. This is the case for both marine hydrothermal vents (shallow and deep-sea vents) and polar (Arctic and Antarctic) areas, where microbial (poly) extremophiles are generally the most abundant life forms [1,2,3,4,5]. Poly-extremophiles are important tools for research in different disciplines, spanning from adaptations to harsh conditions [6], with implications for both the origin of life on Earth [7] and the search for extra-terrestrial life [8,9], to novel strategies to avoid or mitigate contamination for either industrial processes or the astrobiology field [10,11]. Exploring microbial diversity in extreme environments and understanding their adaptive mechanisms allows us to expand our knowledge of potentially habitable environments capable of supporting extraterrestrial life in various planetary bodies within the Solar System, including Mars, Venus, and the satellites of Jupiter (Io, Europa, Ganymede, and Callisto) and Saturn (Titan and Enceladus) [12,13,14,15,16]. Several studies have reported the survival and growth of microorganisms under laboratory-simulated extraterrestrial environments, such as under Enceladus-like and Mars-like conditions [11,17,18].

Resistance to high doses of radiation has been observed in several members of the domains Bacteria and Archaea [19]. Organisms resistant to different types of ionizing radiation (IR; such as X-rays, gamma rays, and charged particles) have been isolated from a number of environments [20], including the radiation resistant thermophile *Deinococcus geothermalis* [21] and the hyperthermophiles *Pyrococcus furiosus* [22], *Thermococcus gammatolerans* EJ [23], and *Thermococcus radiotolerans* [24], which all provided new insights into the field of radiation microbiology.

Members of the *Bacillus* genus are able to produce spores that represent dormant forms that are resistant to several environmental and laboratory stresses, including sterilization techniques such as chemical oxidizing agents, extreme desiccation, wet and dry heat, ultraviolet radiation, and gamma irradiation [25]. Therefore, spores have been acknowledged as the hardiest known form of life on Earth [18]. Bacilli thriving in multi-extreme environmental conditions have been reported to possess high and unexplored resistance to stresses [4,5]. Several novel thermophilic *Bacillus* and *Geobacillus* strains were isolated from shallow hydrothermal vents (SHV) off the Eolian Islands (Italy) and investigated for their potential biotechnological applications [26,27,28,29]. Recently, the spore resistance to artificial and environmental stressors of *Bacillus horneckiae* SBP3 DSM 103063 and *Bacillus oceanisediminis* APA DSM 103062, isolated from two vents off Panarea Island (among the Eolian Islands), has been compared to that of *Bacillus* spp. (*Bacillus horneckiae* DSM 23495, *Bacillus pumilus* SAFR-032, and *Bacillus nealsonii* DSM 15077) isolated from spacecraft assembly facilities, and of *Bacillus subtilis* 168, the biodosimetry strain and space microbiology model organism [30]. Interestingly, spores from SBP3 showed a high level of resistance to stresses that they have never naturally encountered before, such as UV-C, X-rays, hydrogen peroxide, dry heat, and space vacuum. In comparison with *B*. *subtilis* 168, SBP3 spores were significantly more resistant to low-pressure argon plasma, H_2_O_2_, and dry and wet heat [30]. Furthermore, the UV-C and the thermal resistance of spores of *Bacillus licheniformis* T14, isolated from a vent off Panarea Island [28], were also reported under wet heat and dry heat conditions [31,32]. Poly-resistant psychrotolerant *Bacillus* strains, isolated from the active layer in a continuous permafrost area at Edmonson Point (Antarctica) (namely, A30, A34, A43, A45, B51, and B58), were screened for their resistance to several stresses. Spores of A34 and A43 were reported to possess a high degree of resistance to UV-C [31]. Altogether, these results suggested that spores are not necessarily radiation-resistant, but the mechanisms involved with protecting themselves in hot and cold environments could also be involved in resisting the effects of UV-C radiation. Moreover, strains belonging to the same genus or species responded differently to UV-C radiation, which may reflect the adaptation of the studied strains to their own harsh environmental conditions. Thus, isolates from Eolian SHV and Antarctic permafrost active-layer could represent a richer source of poly-resistant bacteria than their mesophilic, terrestrial counterparts.

Survival of bacilli species depends on the ability of dormant spores to grow and multiply under favorable environmental or nutritional conditions. It is known that spore populations vary in the degree of dormancy, with some spores being more dormant than others [33]. Germination can be induced by different types of agents, such as high pressures [34]; temperatures; salinity stresses [35,36]; and nutrient germinants including amino acids, sugars, or purine nucleosides. These agents are involved in a sequence of events that result in a breakdown of the spore structure and the loss of their resistant properties. L alanine, L-asparagine, and L-valine are examples of nutrient germinants binding in a stereospecific manner to spore-specific protein complexes in the inner membrane, termed germinant receptors, and are reported to trigger germination in *B*. *subtilis* [37]. Consequently, the germination process is relevant for scientific investigations (such as in food contamination and disease pathogenesis) to prevent spore germination or to accelerate it and then kill the newly sensitive germinated spores.

One of the main astrobiological goals is the devotion to understanding the limit of microbial resistance to extraterrestrial conditions, with much attention being paid to cosmic radiation as the environmental space parameter that may prevent the preservation and spread of life outside the Earth. The exposure to ionizing radiations, which represent a major part of the cosmic radiation spectrum, can cause cellular damages directly by affecting biomolecules or indirectly by causing radiolysis of water to reactive oxygen species (hydroxyl radicals, superoxide, and hydrogen peroxide) involved in severe oxidative stress to all cellular macromolecules [38]. High (H) Charge (Z) Energy (E) (HZE) particles make up about 1% of the galactic cosmic radiation spectrum and they can reach very high energies, up to 1000 GeV [39,40]. Due to their high ionization energy and great penetration depth, the HZE particles represent one of the main hazardous components of space for any biological system staying for extended periods of time [10,41]. The biological effects of IR are related to the linear energy transfer (LET), the value indicating the local energies transferred by one particle, usually expressed in keV/μm, which is related to a particle’s charge and velocity (energy per nucleon) and the elemental composition of the traversing medium. Therefore, HZE particles’ effects on biological samples depend on their LET values [42,43,44].

In recent times, the STARLIFE international campaign has aimed to study the effects of IR, including those caused by HZE particles in astrobiological model systems, such as spores of *B. subtilis* 168 [45]. Several studies on the survival of *B. subtilis* to high-energy charged particles have reported that the spore’s structural components (i.e., α/β-type small acid soluble spore proteins, core water content, and dipicolinic acid) play an important role in spore resistance, mainly depending on the ability to repair and protect DNA [46,47,48]. Moreover, spore survival depended on the LET of the applied species of ions and radiation, whereas the exposure to HZE particles, for example iron ions (200 keV/µm, high-LET radiation), led to a low level of spore survival compared to low-energy charged particles, such as helium (2.2 keV/µm) [14]. However, there are no studies evaluating the effects of HZE particles on spores from poly-extremophilic bacilli, which may have a different resistant mechanism due to the structural spore’s components, nor on the germination process after HZE exposure.

In this study, the resistance to HZE was investigated on spores from *B*. *horneckiae* SBP3 and *B*. *licheniformis* T14, isolated from Eolian shallow hydrothermal vents, and two *Bacillus* sp. strains A34 and A43 from the Antarctic permafrost active layer, as these strains are highly resistant to environmental and artificial stressors, including non-ionizing radiation (i.e., UV-C). In the frame of the STARLIFE program, we irradiated spores with accelerated heavy ions of helium (He) and iron (Fe) at LET of 2.2 and 200 keV/µm, respectively. The effects related to irradiation with ion species of He and Fe on structural components of spores from thermophilic and psychrotolerant strains were investigated using a spectroscopic FTIR technique. The roles of D-glucose, L-alanine, and L-valine as germinant agents were evaluated in the germination process of spores surviving irradiation.

## 2. Materials and Methods

### 2.1. Sampling Areas

Shallow hydrothermal vents (SHV) of the Eolian Islands (Italy) are characterized by unusual field conditions (high temperature and salinity, low pH values, high concentrations of H_2_S, hydrocarbons, heavy metals, etc.) prohibitive for most organisms [27].

In extremely cold environments, such as those at Edmonson Point on the eastern slope at the foot of Mount Melbourne (Northern Victoria Land), the soils are affected by permafrost, and in their upper part (called the active layer), the microorganisms are able to survive prolonged subzero temperatures (at least 340 days per year); strong fluctuations of temperature, water, and nutrients; and long-term background radiation exposure [49].

### 2.2. Bacterial Strains and Spore Purification

Bacillus strains used in this study are listed in Table 1, together with their optimal growth temperatures and spore resistance to UV-C, expressed as the lethal dose required to kill 90% of the spore population (LD90). Two thermophilic strains, *Bacillus horneckiae* SBP3 [30] and *Bacillus licheniformis* T14 [28] were isolated from samples collected in June 2006 in the immediate vicinity of two shallow submarine hydrothermal vents off Panarea Island (Eolian Islands, Italy), named Black Point (coordinates: 38°38′23″ N–5°06′28″ E, depth 23 m) and Bottaro (coordinates: 38°38′31″ N–15°06′597″ E, depth 8 m). Thermophilic isolates were routinely maintained on tryptone soy agar (TSB; Oxoid, Milan, Italy) supplemented with 1% NaCl (TSA1).

Two psychrotolerant strains, *Bacillus* sp. A34 and A43, were isolated in January 2014 from the Antarctic permafrost active layer at Edmonson Point (coordinates: 74°19′44.2″ S–165°07′59.7″ E, Northern Victoria Land, Antarctica) [49]. *Bacillus* sp. A34 and A43 belong to the Italian Collection of Antarctic Bacteria of the National Antarctic Museum (CIBAN-MNA).

For long term storage, we kept the strains frozen in nutrient broth (Oxoid) at −80 °C with the addition of 50% (v/v) glycerol. 

Sporulation of bacterial isolates was induced in the agarized Schaeffer’s Sporulation Medium (containing 0.1% KCl, 0.012% MgCl_2_, 0.5 mM CaCl_2_, 0.01 mM MnCl_2_, 0.001 mM FeSO_4_, and 1.5% agar) [50]. Overnight cultures in tryptone soy broth (TSB; Oxoid, Milan, Italy) with the addition of 1% NaCl (TSB1) (200 μL) were inoculated by spreading onto the medium, and plates were incubated at each strain’s optimal temperature for 5 days. Spore preparations were checked to be free of vegetative cells and to consist of >99% spores by phase-contrast microscopy. Harvested spores were washed 10 times and resuspended in sterile distilled water before storage at 4°C.

### 2.3. Radiation Exposition to HZE

Preparation of spores for exposure to heavy ion particles was performed as described previously [46]. Briefly, suspensions of spores of the different *Bacillus* strains were prepared in sterile distilled water to a final concentration of 10^8^ spores/mL. Triplicate samples of spore suspensions (100 µL) were individually exposed, between 0 to 8 h, to two different types of high-energy-charged ions: He, with energy of 150 MeV/nucleon (LET = 2.2 keV/µm), and Fe, with energy of 500 MeV/nucleon (LET = 200 keV/µm) (Table 2).

Irradiations were performed at the heavy ion medical accelerator (HIMAC) facility of the National Institute for Radiological Sciences (NIRS) in Chiba, Japan. The irradiation geometry of the HIMAC and dose calculations have been described [51].

### 2.4. Spore Survival

Spore survival was evaluated from appropriate dilutions in distilled water as the ability to form colonies and was expressed as colony forming units (CFU/mL). Aliquots of each sample were inoculated onto plates of TSA1. Plates were incubated at each strain’s optimal temperature for growth, for 24 or 48 h for thermophilic and psychrophilic strains, respectively. The surviving fraction of spores was determined from the quotient N/N_0_, where N is the number of CFU/mL of the irradiated spores and N_0_ represents the CFUs of the non-treated spores. Survival curves were obtained by plotting the logarithm of N/N_0_ as a function of the treatments. Spore inactivation curves were obtained as described previously [51]. Each experiment was repeated three times and the data are expressed as averages ± standard deviations.

In order to compare the resistance of spores from the tested strains with that of *B. subtilis* 168, we elaborated data as D_37_ values, the dose of ionizing radiation required to kill 63% of the initial spore population, determined from the linear portion of the semi-logarithmic curve [46].

The significant differences in the survival rates were determined by analysis of variance (ANOVA) and differences with *p* ≤ 0.05 were considered statistically significant.

### 2.5. Spectroscopic Analysis

Fourier transform infrared (FTIR) spectroscopy was used to identify functional groups, such as carboxyl, phosphate, and amine groups, through their characteristic absorption bands in defined regions of the spectrum [52,53].

The aliquots of spore suspension (20 µL) of non-irradiated (1 × 10^8^ CFU/mL) and irradiated spores were dried in sterile slides at room temperature and then analyzed. Conformational changes in proteins and internal vibrational modes of non-irradiated, He-irradiated, and Fe-irradiated spores were determined by FTIR microscope LUMOS. The spectrometer allowed for the collection of spectral data at 4 cm^−1^ resolution over 128 scans in the 400–4000 cm^−1^ spectral range. To compare the spectra, we normalized them as described previously [53].

### 2.6. Germination Assay

Germination conditions of non-irradiated and He/Fe-irradiated spores were evaluated in the presence of germinant agents D-glucose (Glu), L-alanine (Ala), and L-valine (Val). The germination assay was carried out in 96-well microplates. Germination medium contained 25 mM 4-(2-hydroxyethyl)-1-piperazineethanesulfonic acid (HEPES) buffer (Sigma-Aldrich) (pH 7.4) and 50 mM of each germinant agent. D-glucose (Glu), L-alanine (Ala), and L-valine (Val) were separately dissolved in Milli-Q and filter sterilized through a 0.22 µm pore-size membrane. Wells were filled with 180 µL of germination medium. Each spore suspension in HEPES buffer was added (20 µL) into wells, in 4 replicates, with OD_600nm_ = 0.2. The microplates were incubated at the optimal growth temperature for each strain in a multiplate reader (Thermo Scientific Multiskan GO) and germination was monitored by reading the drop of absorbance at regular intervals (2 min) for 120 min. Microplates were shaken for 5 s prior to each reading.

During germination, the dormant spores lose their refractivity to phase-contrast microscopy. The refractivity loss was quantified spectrophotometrically by measuring the optical density of the germination culture at 600 nm (OD_600nm_) [50]. All OD_600nm_ data were normalized to the starting OD_600nm_—the OD_600nm_ at each measured time point was dived by the first reading (*t*_0 min_), yielding the relative OD_600nm_, given as a percentage (% OD_600nm_). An approximately 60% decrease in the relative OD_600nm_ indicates that all spores in the germination culture have germinated successfully [35]. The lag-time (min), representing the first time-point of the linear part of the decrease in relative OD_600nm_, indicates the duration of the phase before the majority of spores lose their refractivity.

The significance of differences in the germination process was determined by analysis of variance (ANOVA), and differences with *p*-values of ≤ 0.01 or *p*-values ≤ 0.05 were considered statistically significant.

## 3. Results

### 3.1. Spores Survival

**Figure 1:**
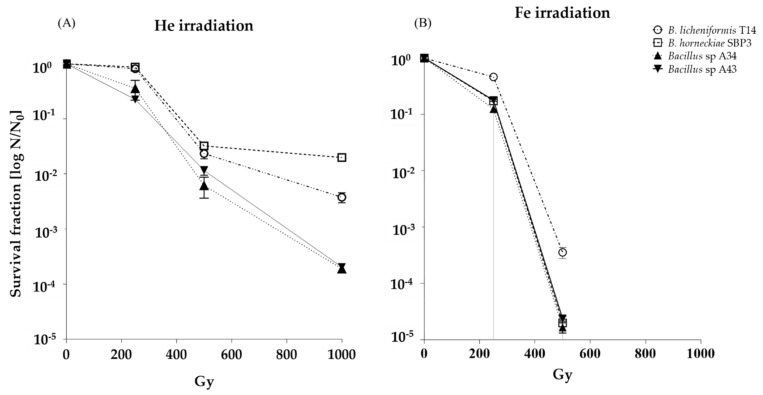
Survival curves of spores from the different tested *Bacillus* spp. to radiations of (**A**) helium ions and (**B**) iron ions.

Survival curves of tested spores from *Bacillus* spp. strains irradiated with He and Fe particles are reported in Figure 1. Each strain’s spore viability, following HZE treatments, showed a decrease in viable spore numbers (N/N_0_, where N is the number of CFU/mL of the irradiated spores and N_0_ represents the CFUs of the non-treated spores) with increasing doses of He and Fe beams (Figure 1).

After exposure to He, inactivation kinetic curves of spores from psychrotolerants exhibited an exponential shape, whereas those from thermophiles showed a sigmoidal shape (Figure 1A). Spores were resistant to He irradiation until 1000 Gy, with thermophiles SBP3 and T14 being more resistant (LD_90_ = 404 ± 12 and 386 ± 18 Gy, respectively) than psychrotolerants A34 and A43 (LD_90_ = 332 ± 15 and 346 ± 12 Gy, respectively); the thermophilic SBP3 strain showed the most robust spore resistance to irradiation with He ions (Figure 1A). The psychrotolerant A34 and A43 strains had the greatest loss of spore viability (about four orders of magnitude).

All spores were shown to survive Fe irradiation until to 250 Gy, whereas at 500 Gy, the spore’s viability from all strains was reduced fivefold, with the only exception being the T14 spores (LD_90_ = 339 ± 21 Gy), for which viability was reduced threefold (Figure 1B).

### 3.2. Spectroscopic Analysis

FTIR analysis was used to determine structural and biochemical changes occurring in spores from bacilli after exposure to He and Fe irradiations in comparison with untreated spores. The peak wavenumbers were assigned according to Vongsvivut et al. [54] Table 3.

Non-irradiated spores from thermophilic and psychrotolerant strains showed major differences in the regions ≅ 780–1200 cm^−1^ (attributed to nucleic acids and carbohydrates), and 1660–1628 cm^−1^ (amide I) and near 1548 cm^−1^ band (amide II), attributed to amino acids and polypeptides, whereas they were more similar in the region 3200–3300 cm^−1^ (amide A). Differences between the spectra of thermophiles, as well as between those of psychrotolerants, were observed mainly in the 800–1200 cm-1 region attributed to carbohydrates.

**Figure 2:**
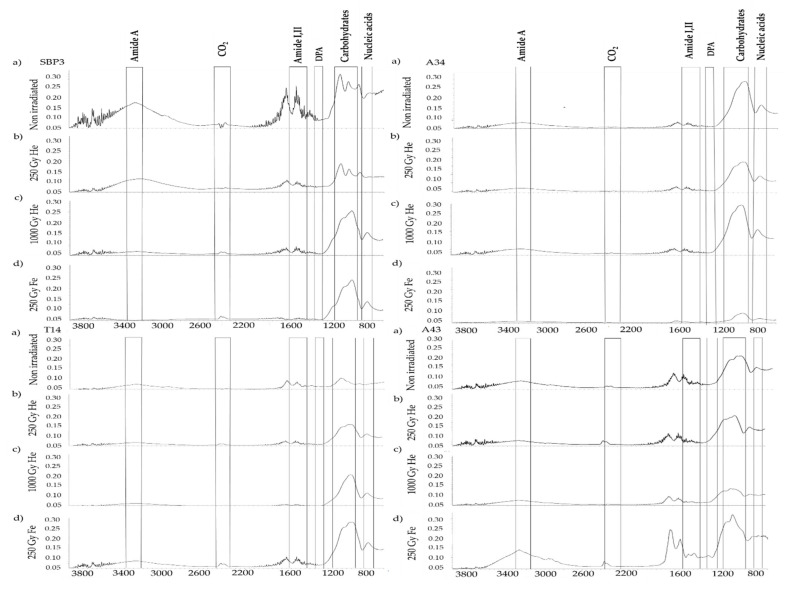
FTIR spectra of spores from *Bacillus horneckiae* SBP3, *Bacillus licheniformis* T14, *Bacillus* sp. A34, and *Bacillus* sp. A43 non-irradiated (**a**) and irradiated with He 250 Gy (**b**), He 1000 Gy (**c**), and Fe 250 Gy (**d**).

Irradiations with He 250 Gy and He 1000 Gy induced shifts and changes in the spectra in a dose-dependent manner (Figure 2). Overall, after He ion irradiations, qualitative changes in the spectra were observed in the 3200–3300 cm^−1^ region, attributed to amide A, with the highest variations observed in the spectrum of A43, and the lowest in T14. In the spectra of irradiated A34 and A43 spores, the peaks resulting in the 2100–2300 cm^−1^ region changed, and their intensity increased, indicating the degradation of lipids with the formation of CO_2_ and CO [63]. The region 1300–1400 cm^−1^, including dipicolinic acid (DPA) peaks, was shown to be reduced in intensity and modified after irradiation with He 1000 Gy and Fe 250 Gy in all strains.

We observed the major effects in the spectrum of A43 after He ion irradiation at 1000 Gy, with evident shifts of peaks in the amide I and II region and in the ≅ 780 cm^−1^ band (attributed to nucleic acids). At the highest dose of He irradiation, we observed evident shifts and changes in the SBP3, T14, and A43 spectra in the region referred to carbohydrates.

After Fe ion irradiation, changes of peaks in SBP3, A34, and A43 spectra were observed in all the regions and referred to as amide I, amide II, DPA, amide A and nucleic acids, lipids, and carbohydrates (800–1200 cm^−1^), whereas the T14 spore spectrum showed minor variations (Figure 2). Modifications induced by Fe 250 Gy irradiation were similar to those observed after exposure to He 1000 Gy.

### 3.3. Germination Assay

**Figure 3:**
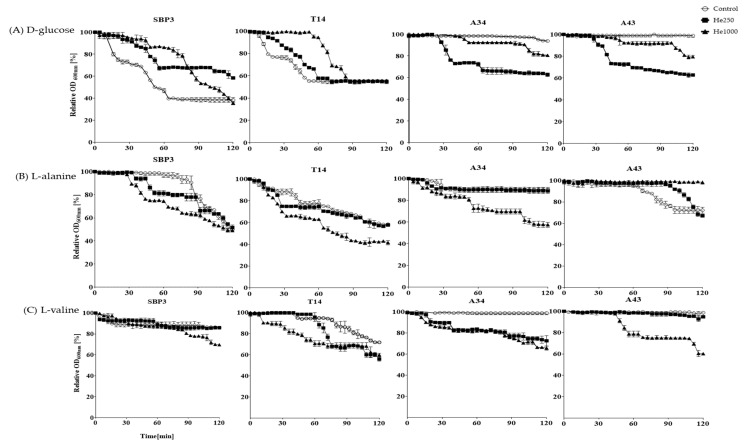
Germination curves of spores non-irradiated (white circles) and irradiated with He 250 Gy (black squares) and He 1000 Gy (black triangles) in the presence of the germinant agents: (**a**) D-glucose, (**b**) L-alanine, and (**c**) L-valine. Germination was monitored as relative absorbance percentage (OD_600nm_%) at regular intervals (2 min) for 120 min of exposure to 50 mM germinant compound.

**Figure 4:**
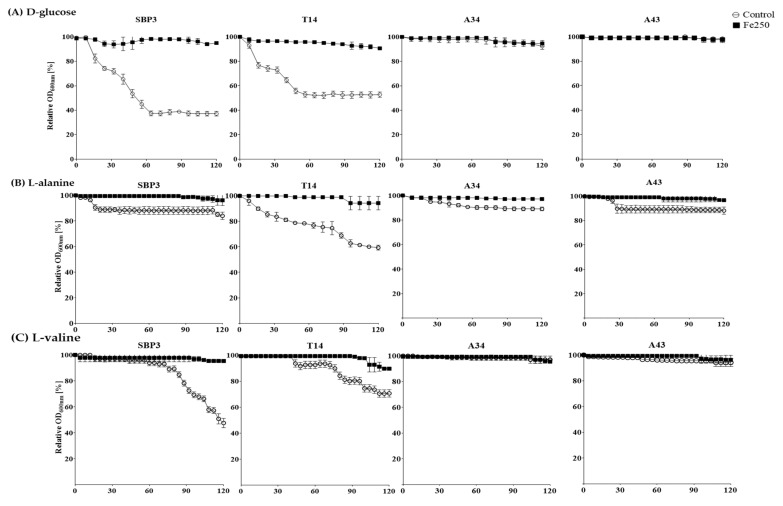
Germination curves of spores non-irradiated (white circles) and irradiated with Fe 250 Gy (black squares) in the presence of the germinant agents: (**a**) D-glucose, (**b**) L-alanine, and (**c**) L-valine. Germination was monitored as relative absorbance percentage (OD_600nm_%) at regular intervals (2 min) for 120 min of exposure to 50 mM germinant compound.

The germination monitoring of non-irradiated and He/Fe ion-irradiated spores by measurement of relative absorbance (% OD_600nm_) at regular intervals (2 min) for 120 min of exposure at each germinant are reported in Figure 3 and Figure 4. The initial loss of absorbance (lag-time) and the germination efficiency, expressed as the relative absorbance after 120 min (% OD_600nm_) in the presence of the different germinant agents as trigger compounds, are reported in Table S1. Overall, germination efficiency varied from ≈1 to 64% within 120 min after the addition of the germinant (Table S1). The ability of spores to germinate was stated as high if the efficiency was 30% or more, and as poor if the efficiency was 10% or less. 

Non-irradiated spores from thermophilic strains germinated in the presence of D- glucose or L-valine as a single germinant compound (Figure 3). Differently from SBP3, spores from T14 were also able to germinate in the presence of L-alanine (Figure 3). A34 spores did not exhibit germination ability with all the germinants tested, whereas A43 spores germinated only with L-alanine (Figure 3). Germination efficiency of non-irradiated spores in the presence of the three used germinants was higher in the thermophilic SBP3 and T14 strains than in the psychrotolerant A34 and A43 spores (efficiency < 30%) (Table S1, Figure 3 and Figure 4).

He irradiation differently affected spore germination at different extents, depending on the dose of irradiation. After He irradiation at 250 Gy, in the presence of D-glucose, the lag-time of thermophiles increased, and therefore germination was much slower than in non-treated spores (Table S1, Figure 3). Interestingly, germination of psychrotolerant A34 and A43 spores was induced with D-glucose after He expositions at 250 Gy (Figure 3). In the presence of amino acids, the lag-time of all spores was shorter, with the only exception being L-alanine for A43 spores (Table S1). After exposure to the maximum dose of irradiation with He ions (1000 Gy), all spores germinated slower with D-glucose than spores irradiated at 250 Gy dose (Figure 3). In the presence of alanine, germination enhanced in the respect of both non-irradiated and irradiated spores at He 250 Gy, with the only exception being spores from A43, which were blocked in germination. In presence of L-valine, all spores exhibited an increased germination, including spores from A43. In general, germination efficiency greatly increased after exposure to He 1000 Gy, with SBP3 and T14 being more efficient (34–64% loss of OD_600nm_) than A34 and A43 (5–47% decrease OD_600nm_) (Table S1). 

Irradiation with Fe 250 Gy inhibited the germination of all spores despite the spores being viable (Figure 1) in the presence of all the trigger agents (Figure 4), indicating a block in spore germination.

## 4. Discussion

High-energy charged particles (HZE), such as protons, neutrons, electrons, and heavy ions, constitute a relevant part of IR by the Sun and galactic sources, or are trapped in the radiation belts of origin. The majority of ionizing radiation particles in space, about 87%, is represented by protons [64]. All planets and celestial bodies are struck by protons and high-charge/high-energy ions, whereas some planets, including the Earth, possess a magnetosphere that traps the majority of ionizing particles. The ice moons Europa and Enceladus possess a magnetosphere from the nearby planets Jupiter and Saturn, respectively, but the impact of the HZE particles that do reach their ice surface produces free radical species that could kill dormant microorganisms [65]. However, environments associated with submarine hydrothermal systems on Earth have recently been observed in Europa and could allow for the development of life under the surface, where the radiation levels are lower [66]. Biological effects of HZE may affect biodiversity since surface radiation contributes to mutagenesis rates, including replication errors and DNA damage from UV, chemical oxidants, and desiccation [67,68]. Deposited energy excites electrons within biomolecules and induces direct damage, whereas the indirect mechanisms of radiation damage are linked to ionization or radiolysis of water, which composes 70–90% of vegetative cells’ weight and 20–40% of bacterial spores’ weight [18]. Water irradiated with IR results in the production of hydrated free electrons and highly reactive oxygen species with unpaired electrons, as well as free radicals, such as H and OH, or their recombination products, such as hydrogen peroxide [69]. These radiogenic products are freely diffusible and migrate from their site of production toward oxidize biomolecules such as DNA and proteins. The presence of oxygen dissolved in irradiated water increases the production of hydrogen peroxide and the hydroxyl radical, and consequently, the indirect damages of macromolecules are greater [70]. The potential damages of the low linear energy transfer (LET) radiation can be attributed to ionization and excitation processes or to the interactions with reactive oxygen species (ROS) [71,72]. ROS could destroy or damage spore coats, core membranes, and/or essential germination receptors, leading to a blockage in spore germination and outgrowth [25,73,74]. Spores of *Bacillus subtilis* are widely studied for their ability to withstand desiccation and low temperature, and therefore they have been used as a model because it is foreseen that even more radiation-resistant bacteria could exist in the subsurface oceans of the icy moons Europa and Enceladus [75]. It is known that spore-protective strategies include (i) the production of two thick and highly cross-linked proteinaceous spore-coat layers [76], (ii) the production and deposition of a melanin-like UV-protective pigment in the spore-coat layer [77], (iii) the dehydration of the spore core via an unknown mechanism involving the spore-cortex protein DacB [78], (iv) the production and storage of large quantities of the calcium chelate of dipicolinic acid (Ca-DPA) in the spore core [79,80], and (v) saturation of spore DNA with α/β-type small acid soluble proteins (SASP) [18,25,81]. Structural differences in the integuments (exosporium, external coat, coat, and inner membrane) could be important in the resistance to HZE exposure of spores [82]. These layers differ among the different species, the spores of *Bacillus horneckiae*, *Bacillus pumilus*, and *Bacillus cereus* are known to possess an exosporium, while spores of *B*. *subtilis* do not [76,83,84,85]. The spore inner membrane (IM) could be involved in the resistance to several stresses, including UV radiation, charged particles, radical species, and neutral and excited atoms [86].

This work provided new insights into the resistance of extremophilic bacilli spores from hot and cold environments to He and Fe particles and how the ionizing radiation influenced their germination processes. Overall, the spores survived better following He rather than Fe ion irradiation at the maximum dose. When exposed to He irradiation, thermophilic SBP3 and T14 strains showed higher spore viability than those of psychrotolerant A34 and A43 strains. Spores from thermophilic and psychrotolerant strains principally differed in their external structures and fatty acid composition. It is known that thermophilic and psychrotolerant strains evolved structural mechanisms toward environmental multi-stresses, including the fatty acid composition of their cellular membranes and of spore IM [87]. The iso-C15 fatty acid content tends to dominate in thermophilic vegetative cells, as previously reported for strains SBP3 and T14 [28,30], while anteiso-C15 fatty acids dominate in psychrophilic bacterial species [88].

**Figure 5:**
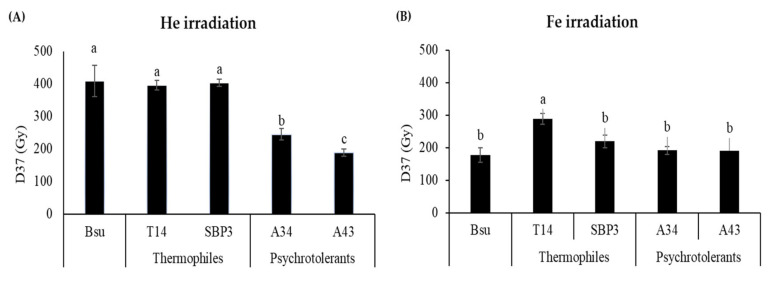
Spore inactivation, expressed as D_37_ values (Gy) after exposure to heavy ions of (**A**) helium (He) and (**B**) iron (Fe) of environmental thermophiles *Bacillus horneckiae* SBP3 and *Bacillus licheniformis* T14, and psychrotolerants *Bacillus* sp. A34 and A43, with comparison to *Bacillus subtilis* 168 (Bsu, data from Moeller et al. [47]). Resistance of spores is expressed as averages and standard deviations (*n* = 3). For each graph, the lowercase letters above the bars denote groups with significant differences by ANOVA (*p* ≤ 0.05).

To compare the spore resistance to He and Fe ions of extremophilic strains with that *B. subtilis* 168 (Bsu), we expressed data as the dose of ionizing radiation killing 63% of the initial spore population (D_37_ values) (Figure 5).

The SBP3 and T14 spore resistance to He ions was similar to spores of *B*. *subtilis* 168 (Figure 5A), which are considered powerful dosimeters for terrestrial environmental monitoring and extraterrestrial studies. Spores of T14 had the greatest resistance to Fe nuclei, which are known to present more lethal effects to spores than He nuclei [47], whereas spores of the other strains demonstrated equal resistance as *B*. *subtilis* 168 (Figure 5B).

The effects of He and Fe accelerated ions on the survived spores from thermophilic and psychrotolerant strains were investigated on structural components by using a spectroscopic technique (FTIR). Significative qualitative variations (i.e., number of peaks and their shape) in the spectra of spores were observed at increased dose of He and at Fe 250 Gy irradiation. In comparison with non-irradiated spores after He 250 Gy irradiation, we observed no significative change in all spore spectra, whereas after He 1000 Gy irradiation, the variations in the regions attributed to carbohydrates, lipids, and proteins (amide I and II) were evident in all the spectra, with distinctive changes for each strain. Furthermore, the FTIR spectra indicated that the irradiation with Fe 250 Gy was responsible for large injuries in all spore structures, including DNA, similarly to those observed after irradiation with He 1000 Gy.

Moeller et al. [89] reported that after He and Fe ion irradiation, the spores of *B*. *subtilis* possessed the following repair DNA mechanisms: (i) apurinic/apyrimidinic endonucleases, and (ii) non-homologous end joining repair-deficient. The α-β- SASP mutants were subjected to a higher rate of mutation. SASPs play essential roles in the prevention of both lethal and mutagenic damages to spore DNA induced by He and Fe ion irradiation [82,89]. Therefore, He and Fe particles, as producers of oxidizing agents by radiolysis of water, could be responsible of the damages in the structural spore components. However, further investigation is needed to determine the structural (i.e., protein, enzyme, or lipidic/carbohydrate coat) and molecular differences, such as the presence and efficiency of the DNA repair system involved in the spore resistance to HZE particles.

The spores from extremophilic bacteria that survived irradiation showed different germination kinetics, depending on the type/dose of irradiation and the germinant agent used. Although several exogenous germinants are reported to trigger germination in different species of *Bacillus*, different nutrient germinants are species-specific. In this study, D-glucose was the principal germinant agent for non-irradiated spores from thermophilic strains, but not for those from psychrotolerants. After exposure to He ions, the thermophilic spores germinated slower in the presence of D-glucose, probably due to the alteration of one or more germinant receptors (Ger) (e.g., GerA, YndD, or GerK), since none of the GRs can initiate an efficient germination on their own, as reported in spores from *B. subtilis* and *B. licheniformis* strain MW3 [90]. Surprisingly, glucose induced the germination of spores from psychrotolerants after exposition to He, suggesting that the radiation may affect the glucose receptor complex either directly by altering the GR structure and affinity, or indirectly by increasing the IM permeability. Presumably, the irradiation with He ions, inducing damages in the spore cortex structure of psychrotolerants, allowed for the increasing permeability of IM to glucose, as well as to amino acids L-alanine and L-valine, with the only exception being alanine for A43. Moreover, when triggered with L-alanine, the He-irradiated spores of T14 showed an increase in the germination efficiency and a decrease in lag time. This finding is corroborated by previous results indicating that the germination of *B. licheniformis* mutants defecting in the coat assembly increased with L-alanine [91]. Furthermore, the increasing permeability to L-alanine, as reported in germination of *B. subtilis* after heat exposition, was related to the upregulation of GerA and genes associated with L-alanine uptake [92]. Interestingly, all alive spores irradiated with Fe 250 Gy were unable to germinate with the agents used, indicating a block in germination, probably due to the damages of germinant receptor complex.

This study encourages future research to explore the relationship between the environmental conditions, such as temperature, and the radio-resistant properties of spores from extremophiles.

## 5. Conclusions

The HZE ions irradiation resistance of poly-extremophilic bacilli spores from hot and cold environments was investigated, resulting in modifications to structural components of spores and how those modifications influence the germination process.

Spores survived He irradiation until the highest dose, whereas they were drastically reduced at Fe ions, which are known to present more lethal effects to spores than He nuclei. Spores from thermophiles survived irradiation better than psychrotolerants, and T14 spores possessed the greatest resistance to Fe nuclei.

Spores that survived after irradiation showed different germination kinetics, depending on the type/dose of irradiation and the germinant agent used. After He irradiations, spores germinated more slowly in the presence of the appropriate germinant agent, as was the case for thermophiles in the presence of D-glucose. Paradoxically, after He radiation, spores’ germination efficiency increased, or spores even germinated with agents that in non-irradiated conditions were not efficient. This phenomenon was most evident in psychrotolerants and can be considered as the main effect of radiation damage on the structures of spores, mainly on proteins involved in spore germination, which resulted in the increased permeability of the internal membranes and also in the alteration in the structures of the receptor complex. All spores were blocked in germination after irradiation with Fe 250 Gy. The molecular effects of the radio-activation of spores are not as well known; therefore, further studies are needed to define the molecular mechanisms involved in the germination of our extremophiles after irradiation.

Our findings provide novel insights on the effects of HZE particles on viability and germination process of spores from poly-extremophilic bacilli. The knowledge of germinant agents and the time of out-growth could help us to determine correctly, rather than underestimate, the viability of dormant spores in different contexts, including astrobiology and the planetary protection context.

## Figures and Tables

**Table 1:** *Bacillus* species used in this study, their optimal temperature for growth (°C), and their spore resistance (LD_90_) to UV-C (254 nm).

Strain	Source	Optimal Temperature for Growth (°C)	Spore Resistance to UV-C (LD_90_)	Reference
*Bacillus horneckiae* SBP3	Black point, shallow hydrothermal vent (Panarea Island, Italy)	45	139 ± 15	[30]
*Bacillus licheniformis* T14	Bottaro, shallow hydrothermal vent (Panarea Island, Italy)	50	127 ± 13	[31]
*Bacillus* sp. A34	Antarctic soil (Edmonson point)	15	127 ± 10	[31]
*Bacillus* sp. A43	Antarctic soil (Edmonson point)	15	110 ± 10	[31]

**Table 2:** Radiation used in STARLIFE experiments.

Ion	Energy (MeV/n)	LET (keV/µm)	Intensity (Particles/s)
Helium (He)	150	2.2	2.0 × 10^9^
Iron (Fe)	500	200	2.5 × 10^8^

**Table 3:** FTIR band assignments for the functional groups.

Wavenumber Values (cm^-1^)	Band Assignment	References
3300–3200	H-bond and OH group of alcohol, phenols, and organic acid including nucleic acids and proteins amide A	[55]
3100–3000	n(C–H) of cis C=H bonds	[56]
≈2925	(C–H) from methylene (–CH) group of lipids	[56]
1700–1750	Protein and esters of muramic acid and ester fatty acid group	[56]
1660–1628	Amide I peptidic conformation	[57]
1548	Amide II peptidic conformation	[58]
≈1380	CH_2_ and CH_3_ bending from lipids, DPA	[59]
≈1310	amide III	[60]
≈1066	(R–O–P–O–R) from ring vibrations of carbohydrates	[56]
≈966	(CH) of conjugated trans, trans isomers	[61]
≈780	Nucleic acids, sugar-phosphate vibration	[62]

Spectra of spores from bacilli display all the characteristic regions associated with amide A, amide I, amide II, dipicolinic acid (DPA), carbohydrates, and nucleic acids peaks. The structural similarity and differences between the examined regions can be seen qualitatively in the spectra reported in Figure 2.

## Supplementary Materials

The following are available online at https://www.mdpi.com/2075-1729/10/11/264/s1, Table S1: Germination process of non-irradiated spores and He-/Fe-irradiated spores in the presence of D-glucose (Glu), L-alanine (Ala), and L-valine (Val) as trigger compounds; the lag-time (the initial loss of the relative absorbance) and the efficiency of germination (loss of the relative absorbance percentage, OD_600nm_%) after 120 min of exposure to the 50 mM germinant compound.

## Abbreviations

Ala	L-alanine
AP	Apurinic/apyrimidinic
CFU	Colony forming unit
LD_90_	Lethal dose required to kill 90% of the spore population
FTIR	Fourier transform infrared
GCR	Galactic cosmic radiation
Glu	D-glucose
GRs	Germinant receptors
HIMAC	Heavy ion medical accelerator center
HZE	Heavy charge energy
IR	Ionizing radiation
LET	Linear energy transfer
NHEJ	Non-homologous end joining
NIRS	National Institute for Radiological Sciences
OD	Optical density
ROS	Radical oxygen species
SASP	Small acid soluble proteins
SHV	Shallow hydrothermal vents
TSA	Tryptone soy agar
TSA1	Tryptone soy agar with 1% NaCl
Val	L-valine
