# Supplementary material for: Effects of Heavy Ion Particle Irradiation on Spore Germination of Bacillus spp. from Extremely Hot and Cold Environments
Source: Life (Basel). 2020 Oct 30;10(11):264. doi: 10.3390/life10110264 (PMC7693761; doi:10.3390/life10110264)
Supplement: Supplementary file 1 [file life-10-00264-s001.pdf]

Supplementary Material

Effects of Heavy Ion Particle Irradiation on Spore Germination of *Bacillus* spp. from Extremely Hot and Cold Environments

Table S1. Germination process of non-irradiated spores and He-/Fe- irradiated spores in the presence of D-glucose (Glu), L-alanine, (Ala) and L-valine (Val) as trigger compounds; the lag-time (the initial loss of the relative absorbance) and the efficiency of germination (loss of the relative absorbance percentage, OD_{600nm}%) after 120 min of exposure to 50 mM germinant compound.

Strain	Non-Irradiated Spores			He Irradiated Spores 250 Gy			He Irradiated Spores 1000 Gy			Fe Irradiated Spores 250 Gy		
	Trigger	Lag-Time (min)	Efficiency $_{T120}$ (%OD_{600nm})	Trigger	Lag-Time (min)	Efficiency $_{T120}$ (%OD_{600nm})	Trigger	Lag-Time (min)	Efficiency $_{T120}$ (%OD_{600nm})	Trigger	Lag-Time (min)	Efficiency $_{T120}$ (%OD_{600nm})
B. horneckiae SBP3	Glu	16	60.8 ± 1.1	Glu	38*	50.5 ± 1.1	Glu	82*	64.4 ± 1.6	Glu	40	4.5±1.1
	Ala	82	45.7 ± 1.8	Ala	53*	49.4 ± 0.9	Ala	41*	50.8 ± 1.4	Ala	88	4.7 ± 0.9
	Val	85	18.1 ± 1.1	Val	55*	19.0 ± 1.5	Val	40*	33.6 ± 1.6*	Val	96	5.0 ± 1.1
B. licheniformis T14	Glu	14	44.2 ± 0.9	Glu	28*	44.4 ± 1.0	Glu	70*	44.2 ± 1.1	Glu	16	1.4 ± 1.0
	Ala	80	45.6 ± 4.4	Ala	63	46.3 ± 4.1	Ala	46*	63.4 ± 5.5*	Ala	48	10.3 ± 0.9
	Val	86	30.1 ± 2.4	Val	64*	47.5 ± 3.2*	Val	45*	49.4 ± 6.5*	Val	96	10.5 ± 1.1
Bacillus sp. A34	Glu	27	0.8 ± 1.0	Glu	103*	34.9 ± 1.7*	Glu	117*	24.3 ± 2.3*	Glu	64	4 ± 1.7
	Ala	95	2.4 ± 1.4	Ala	93	0.8 ± 0.03	Ala	23*	39.8 ± 1.4*	Ala	93	0.8 ± 0.03
	Val	120	1.4 ± 1.0	Val	43	27.9 ± 4.4*	Val	39*	34.8 ± 1.3*	Val	108	2.9 ± 0.4
Bacillus sp. A43	Glu	120	8.9 ± 1.5	Glu	42*	37.4 ± 1.1*	Glu	104	30.5 ± 1.3*	Glu	120	1.2 ± 0.1
	Ala	75	25.6 ± 2.6	Ala	97	32.7 ± 0.4	Ala	120	4.9 ± 0.4*	Ala	120	0
	Val	120	3.7 ± 0.6	Val	119	4.2 ± 0.6	Val	56*	46.8 ± 1.2*	Val	100	2.2 ± 0.3

*Significantly different ($p \leq 0.01$) compared with non-irradiated
